# The Sec1/Munc18 Protein Vps45 Regulates Cellular Levels of Its SNARE Binding Partners Tlg2 and Snc2 in *Saccharomyces cerevisiae*

**DOI:** 10.1371/journal.pone.0049628

**Published:** 2012-11-14

**Authors:** Scott G. Shanks, Lindsay N. Carpp, Marion S. Struthers, Rebecca K. McCann, Nia J. Bryant

**Affiliations:** Henry Wellcome Laboratory of Cell Biology, Institute of Molecular, Cell & Systems Biology, College of Medical, Veterinary and Life Sciences, University of Glasgow, Glasgow, United Kingdom; Institute of Molecular and Cell Biology, Singapore

## Abstract

Intracellular membrane trafficking pathways must be tightly regulated to ensure proper functioning of all eukaryotic cells. Central to membrane trafficking is the formation of specific SNARE (soluble N-ethylmeleimide-sensitive factor attachment protein receptor) complexes between proteins on opposing lipid bilayers. The Sec1/Munc18 (SM) family of proteins play an essential role in SNARE-mediated membrane fusion, and like the SNAREs are conserved through evolution from yeast to humans. The SM protein Vps45 is required for the formation of yeast endosomal SNARE complexes and is thus essential for traffic through the endosomal system. Here we report that, in addition to its role in regulating SNARE complex assembly, Vps45 regulates cellular levels of its SNARE binding partners: the syntaxin Tlg2 and the v-SNARE Snc2: Cells lacking Vps45 have reduced cellular levels of Tlg2 and Snc2; and elevation of Vps45 levels results in concomitant increases in the levels of both Tlg2 and Snc2. As well as regulating traffic through the endosomal system, the Snc v-SNAREs are also required for exocytosis. Unlike most *vps* mutants, cells lacking Vps45 display multiple growth phenotypes. Here we report that these can be reversed by selectively restoring Snc2 levels in *vps45* mutant cells. Our data indicate that as well as functioning as part of the machinery that controls SNARE complex assembly, Vps45 also plays a key role in determining the levels of its cognate SNARE proteins; another key factor in regulation of membrane traffic.

## Introduction

Spatial and temporal regulation of membrane traffic is essential for maintenance of one of the defining features of eukaryotic cells; intracellular compartmentalisation into discrete membrane bound organelles [1]. The SNARE (soluble *N*-ethylmaleimide-sensitive factor attachment protein receptor) proteins are central to membrane traffic in all eukaryotes, with formation of SNARE complexes between SNAREs on opposing membranes being defined as the minimal requirement for membrane fusion [2]. SNARE complexes consist of 4 helical SNARE motifs contributed by members of the Qa (syntaxin), Qb, Qc (also called t-SNAREs) and R- (also called v-SNAREs) subfamilies [3].

Regulating SNARE complex assembly allows the cell to regulate membrane traffic. One family of proteins that regulate this process are the Sec1/Munc18 (SM) proteins [4]. Although their mechanism of action is not completely understood, it is clear that SM proteins are universally required for functional SNARE complex assembly [4]. We have previously characterised a role for the SM protein Vps45 in regulating SNARE-mediated membrane traffic through the endosomal system of the yeast *Saccharomyces cerevisiae* [5], [6], [7]. Vps45 activates the syntaxin Tlg2 for entry into functional SNARE complexes with its partner t-SNAREs Tlg1, Vti1 and the v-SNARE Snc2 likely by facilitating a switch of Tlg2 from a closed (inactive) conformation to an open (active) state [5]. Vps45 binds directly to Tlg2, as well as to the v-SNARE, Snc2 and the assembled SNARE complex [6], [7], [8]. Of these interactions, Vps45’s binding to Tlg2 is the most thoroughly characterised. Like other SM proteins Vps45 has two distinct binding sites for its cognate syntaxin [8]. The higher-affinity binding site involves a conserved leucine residue in Vps45 that is predicted to form a hydrophobic pocket on the outer surface of the arch shaped SM protein [7]. This facilitates ‘mode 2’ binding to Tlg2 which requires the N-terminal 36 residues of the syntaxin, and is akin to the interaction captured by the Sly1/Sed5 crystal structure [7], [9]. In addition, Vps45 binds Tlg2 through a second mechanism that does not involve the N-terminal peptide of the syntaxin, and while the structure of this interaction has not been solved, biochemical and biophysical studies indicate that it is akin to the interaction captured in the crystal structure of the neuronal Munc18a bound to Syntaxin1a; mode 1 binding [8], [10]. The role that these two distinct binding modes between Vps45 and Tlg2 and indeed, between other SM proteins and their cognate syntaxins is not understood.

Vps45 was originally identified through genetic screens for yeast defective in vacuolar protein sorting (*vps* mutants) [11], [12], and has been shown to be required for delivery of proteins from the *trans* Golgi network (TGN) into the endosomal system [13], [14]. Classification of the *vps* mutants into 6 groupings (A–F) based on vacuolar morphology suggested the existence of 6 separate processes whose individual perturbation result in missorting of the vacuolar hydrolase carboxy peptidase Y (CPY) [15]. This suggested that there are multiple trafficking steps in the Vps pathway from the TGN to the vacuole, and that gene products affected in mutants of each of the classes act in the same process or membrane trafficking step. Further studies have revealed that this in indeed the case, and in many cases proteins encoded by genes in the same class function as part of a multi-protein complex. This is perhaps best exemplified by the class E *vps* mutants [15]. Class E *vps* mutants accumulate an exaggerated form of the endosomal compartment through which biosynthetic and endocytosed material transits *en route* to the vacuole [15]. Biochemical, morphological and genetic studies have demonstrated that the gene products represented by Class E *vps* mutants all function in sorting ubiquitinated proteins into multivesicular bodies as part of the ESCRT (**e**ndosomal **s**orting **c**omplex **r**equired for **t**ransport machinery [16], [17]. Similarly, the 4 class C proteins (Vps11, Vps16, Vps18 and Vps33) form the core of the CORVET and HOPS tethering complexes that function at endosomal and vacuolar membranes respectively [18]. *VPS45* is a class D gene [15]. Class D Vps proteins, including Vps45 and the regulatory and catalytic subunits of PI-3 kinase (Vps15 and Vps34), are required for traffic between the TGN and endosomes [19].

**Figure 1:**
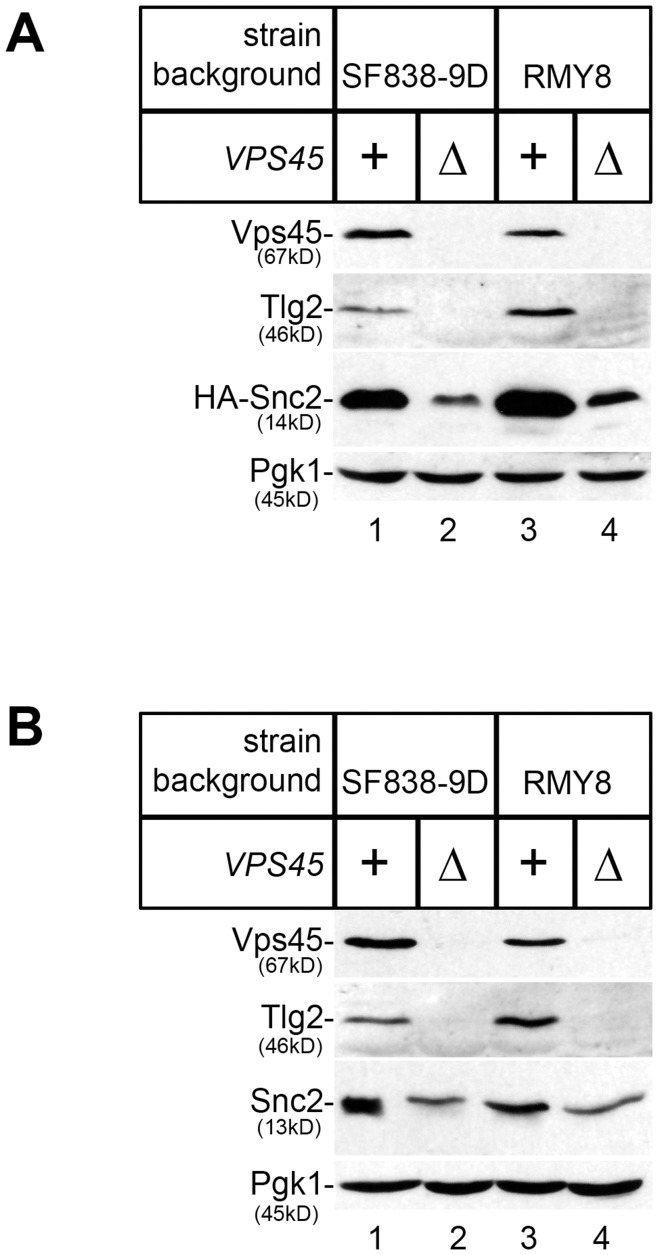
Deletion of VPS45 results in reduced cellular levels of Tlg2 and the Snc2 proteins. Proteins contained within cell lysates prepared from wild-type (SF838-9D and RMY8; lanes 1 and 3) and congenic *vps45*Δ mutant cells (NOzY1 and MSY002; lanes 2 and 4) were separated using SDS-PAGE before being transferred to nitrocellulose. The resulting filters were probed using antibodies that recognise Vps45, Tlg2, the HA-epitope [for HA-tagged Snc2 expressed from pCOG054] in (A), the Snc v-SNAREs in (B), and Pgk1 (phosphoglycerare kinase, which was included as a loading control). N.B. The cells used for (A) harbour pCOG054 whereas those used in (B) do not.

Interaction between Vps45 and Tlg2 is not only important for regulating the function of Tlg2, but also stabilises the syntaxin, with cellular levels of Tlg2 being dramatically reduced in cells lacking Vps45 [5], [20]. This feature appears to be conserved across species, and also across different membrane trafficking steps, as several SM proteins have been shown to stabilise their syntaxins [21], [22], . Controlling cellular levels of SNARE proteins represents another mechanism by which membrane fusion may be regulated. Like many SM proteins, Vps45 also binds directly to its cognate v-SNARE [7], and we therefore set out to investigate whether v-SNARE levels are also regulated by binding to the SM protein. We now report that, like those of Tlg2, levels of the Snc v-SNARE are regulated by Vps45.

**Figure 2:**
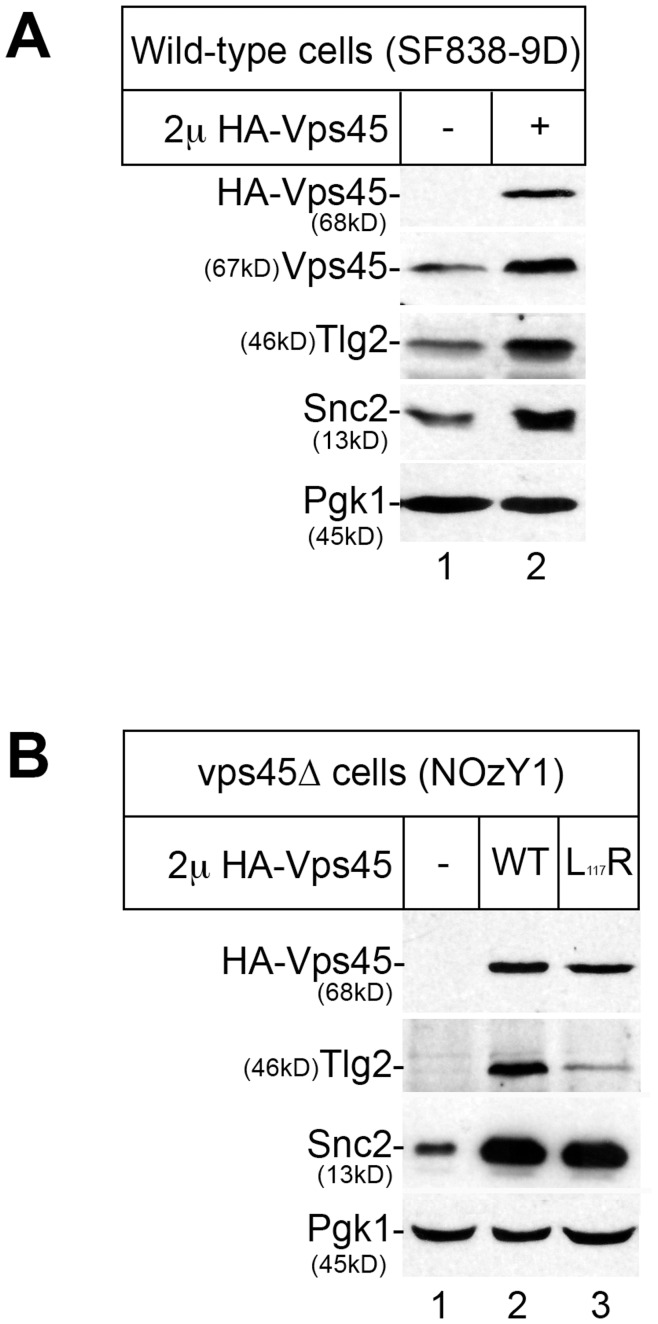
Elevated cellular levels of Vps45 result in a concomitant increase in Tlg2 and Snc2 levels. (A) Proteins contained within cell lysates were prepared from wild-type cells (SF838-9D) harbouring either the empty vector YEplac195 (lane 1) or the multicopy 2 μ plasmid pCOG070 encoding HA-Vps45 (lane 2), were separated using SDS-PAGE before being transferred to nitrocellulose. The resulting filters were probed using antibodies that specifically recognise the HA-epitope, Vps45, Tlg2, Snc2 and Pgk1 as indicated. (B) Proteins contained within cell lysates were prepared as described in (A) from *vps45*Δ mutant cells (NOzY1) producing either no Vps45 (carrying the empty vector YEplac195; lane 1), wild-type HA-Vps45 (lane 2) or HA-Vps45_L117R_ (lane 3) from multicopy 2 μ plasmids (pCOG070 and pCOG071 respectively).

As well as assembling into Tlg2p-containing SNARE complexes to regulate endosomal traffic, the Snc2 v-SNAREs also participate in exocytic Sso-containing SNARE complexes required for fusion of secretory vesicles with the plasma membrane; a trafficking pathway required for growth [25], [26]. These data explain why *vps45*Δ cells are unique amongst the Class D *vps* mutants in that they display multiple growth phenotypes and reveal the importance of regulating cellular levels of SNARE proteins.

**Figure 3:**
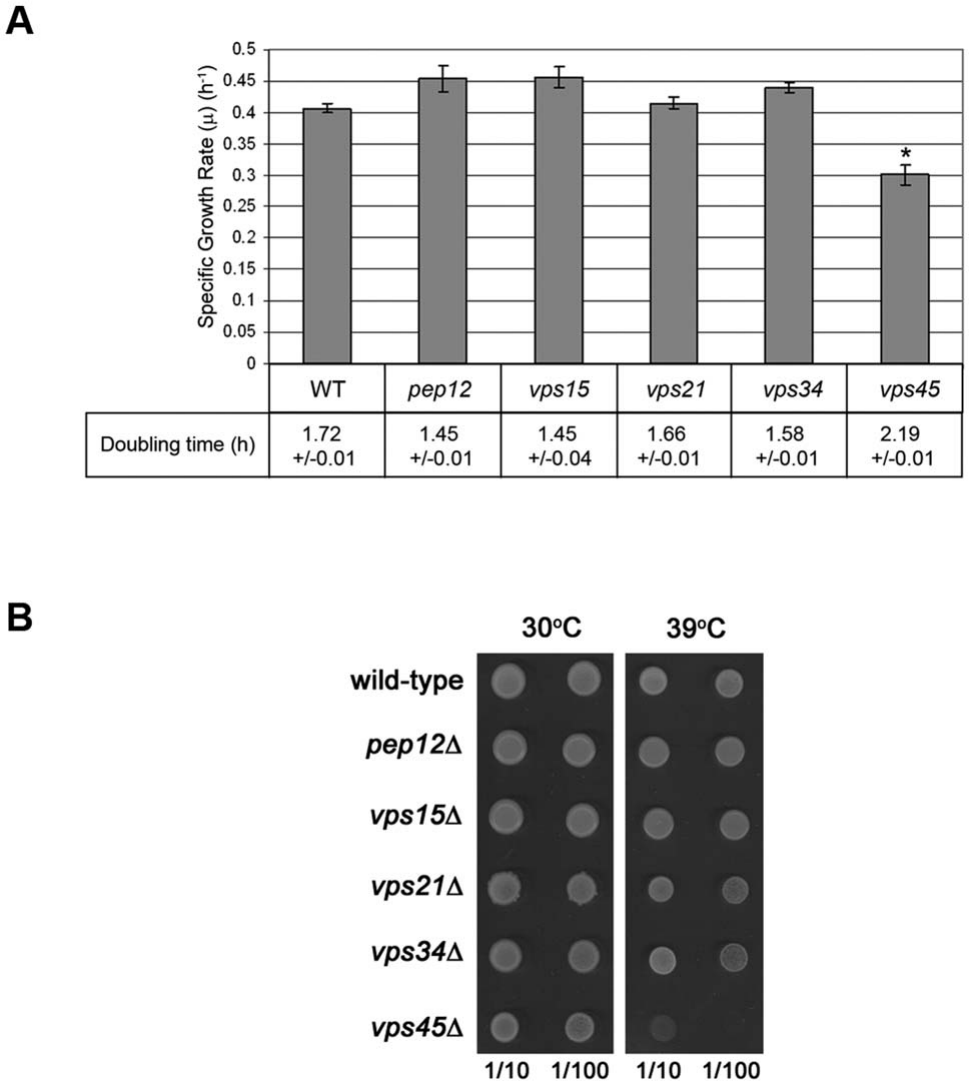
vps45 mutants display growth defects not found in other class D vps mutants. (A) The specific growth rate (μ) and doubling time of wild-type cells (SF838-9D) and congenic class D *vps* mutants (*pep12*, *vps15*, *vps21*, *vps34*, *vps45*) was determined during logarithmic growth in YEPD. Data represent the mean ± SEM of 6 independent sets of cells. *, P<0.05 versus wild-type. (B) Cells were grown for 16 h at 30°C in a shaking incubator, before being diluted to an OD_600_ of 0.2 and grown for 2 further doublings. 10 OD_600_ equivalents of cells were harvested and resuspended in 1 ml dH_2_O. 5 µl of a 1/10 and a 1/100 dilution of this suspension was spotted onto solid media and incubated at either 30°C or 39°C for 3 days.

## Results and Discussion

### Vps45p Regulates Levels of its Cognate SNARE Binding Partners

Cells lacking the SM protein Vps45 have reduced levels of its cognate syntaxin Tlg2 [5], [7], [20], [27]. In addition to binding Tlg2, Vps45 also binds the v-SNARE Snc2 [7]. To address whether absence of Vps45 affects cellular levels of Snc2, we used immunoblot analysis to assess the levels of an HA-tagged version of Snc2 in wild-type and *vps45*Δ mutant cells. Figure 1A shows that cells lacking Vps45 (*vps45*Δ) contain substantially less HA-Snc2 than wild-type cells harbouring the same HA-Snc2 expression construct (pCOG054). Analysis of two different strain backgrounds demonstrates that cellular levels of HA-Snc2 produced from the 2µ-plasmid in *vps45*Δ mutant cells are approximately 33% of those found in their congenic wild-type strains (Fig. 1A). To extend these studies and look at levels of the endogenous v-SNARE, we used polyclonal antiserum raised against residues 72–86 (GFKRGANRVRKQMWW) of Snc2 [28]. This region of the protein shares 87% identity with the analogous region of Snc1 (residues 73–87; GFKRGANRVRKAMWY), whose functions are redundant with Snc2 for cellular processes including exo- and endocytosis [26], [29]. Immunoblot analysis using this antiserum demonstrates that cellular levels of endogenous Snc v-SNAREs in cells lacking Vps45 (*vps45*Δ) are approximately 65% of those in congenic wild-type cells (Fig. 1B).

**Figure 4:**
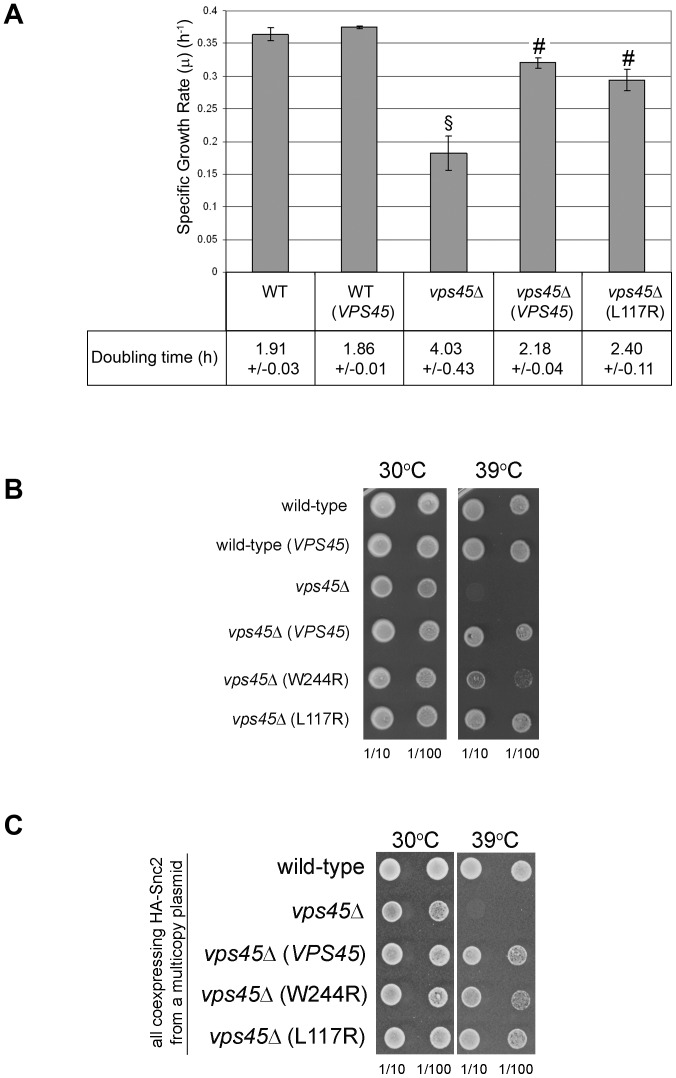
Vps45_L117R_ complements growth phenotypes of vps45Δ cells. (A) The specific growth rate (μ) and doubling time of wild-type (SF838-9D) and congenic *vps45*Δ mutant (NOzY1) cells producing either no HA-Vps45 (carrying the vector YEplac195), wild-type HA-Vps45 (*VPS45*) or HA-Vps45_L117R_ (L117R) from pCOG070 and pCOG071 respectively (WT and *vps45*Δ cells producing no HA-Vps45 carried the vector YEplac195) was determined during logarithmic growth in minimal media. Data represent the mean ± SEM of 6 independent sets of cells. §, P<0.05 versus WT; #, P<0.05 versus *vps45*Δ. (B) Wild-type cells (SF838-9D) harbouring YEPlac195 or pCOG070 (HA-Vps45), and congenic *vps45*Δ mutant cells (NOzY1) harbouring plasmids YEplac195 (empty vector), pCOG070 (HA-Vps45), pCOG71 (HA-Vps45_L117R_) or pCOG072 (HA-Vps45_W244R_) were analysed as described for Figure 3B. (C) All yeast strains in this panel harbour plasmid pCOG054, producing HA-Snc2 in addition to the following plasmids. Wild-type cells (SF838-9D) harbouring YCplac111 (empty vector) and congenic *vps45*Δ mutant cells (NOzY1) harbouring YCplac111 (empty vector) or pNB706 (HA-Vps45), pNB707 (HA-Vps45_L117R_) or pNB708 (HA-Vps45_W244R_) were analyzed as in Figure 3B.

The data presented in Figure 1 indicate that cellular levels of the v-SNARE Snc proteins and the syntaxin Tlg2 are regulated by their cognate SM protein Vps45p. To test this hypothesis we set out to increase the cellular levels of Vps45 and ask whether this leads to a concomitant increase in Tlg2 and Snc levels. This was achieved by expressing *VPS45* from a multicopy plasmid in wild-type cells (Fig. 2A). Cells harbouring pCOG070, encoding HA-tagged Vps45 [7], contain approximately 2.5-fold more Vps45 than those carrying the parental vector YEplac195 [30], and also contain increased levels of Tlg2 and the Snc v-SNAREs (both ≥2-fold).

**Table 1:** Yeast strains used in this study.

Strain	Genotype	Reference
SF838-9D	*MATα ura3–52 leu2–3, 112 his4–519 ade6 gal2 pep4-3*	[12]
NOzY1	*MATα ura3–52 leu2–3, 112 his4–519 ade6 gal2 pep4-3 vps45*Δ*::Kan^r^*	[5]
RMY8	*MATα ura3–52 leu2–3, 112 his3–Δ200 trp1–Δ901 suc2–Δ9 lys2–801 pep4–*Δ*H3*	This study
MSY002	*MATα ura3–52 leu2–3, 112 his3–Δ200 trp1–Δ901 suc2–Δ9 lys2–801 pep4–*Δ*H3 vps45*Δ*::Kan^r^*	This study

RMY8 and MSY002 are congenic to SEY6210 [11]. NOzY1 is congenic to SF838-9D.

**Table 2:** Plasmids used in this study.

Plasmid	Description	Reference
pCOG054	Yeast expression plasmid (2 μ, *URA3*) encoding HA-Snc2	This study
pCOG070	Yeast expression plasmid (2 μ, *URA3*) encoding wild-type HA-Vps45	[7]
pCOG071	As pCOG070, encoding HA-Vps45_L117R_	[7]
pCOG072	As pCOG070, encoding HA-Vps45_W244R_	[7]
YEplac195	Parent vector of pCOG070/1/2	[30]
pNB706	Yeast expression plasmid (CEN, *LEU2*) encoding wild-type HA-Vps45	[7]
pNB707	As pNB706, encoding HA-Vps45_L117R_	[7]
pNB708	As pNB706, encoding HA-Vps45_W244R_	[7]
YCplac111	Parent vector of pNB706, pNB707 and pNB708	[30]

### Selective Stabilisation of Snc by a Mutant Version of Vps45 Abrogated for Tlg2-Binding

We have previously reported that a version of Vps45 (Vps45_L117R_) harbouring a mutation that abrogates binding of the SM protein to the N-terminal peptide of Tlg2 [7], [8] does not stabilise Tlg2, demonstrating that this mode of binding between Vps45 and Tlg2 stabilises the syntaxin [27]. The L117R mutation disrupts the hydrophobic pocket-binding site situated on the outer surface of domain-1 of the arch-shaped SM protein [7], [8]. The binding site for Snc2 is distinct from this hydrophobic pocket, demonstrated by the observation that the Vps45_L117R_ mutant binds the cytosolic domain of Snc2 *in vitro* in a manner indistinguishable from wild-type Vps45 [7]. Figure 2B shows that, as previously reported [27], *vps45*Δ cells expressing Vps45_L117R_ have reduced levels of Tlg2 compared to the same cells expressing wild-type Vps45. In contrast, Snc levels are comparable in *vps45*Δ cells expressing either Vps45_L117R_ or wild-type Vps45 (both substantially higher than in the same cells carrying empty vector; Fig. 2B). These data indicate that binding to Vps45 is important for the regulation of cellular levels of its cognate SNARE binding partners. This is an important observation as it provides the cell with a mechanism to ensure that the SNAREs are not present in excess compared to their regulatory SM protein. Were such a situation to arise it could prove catastrophic for the cell allowing SNARE complexes to form in an unregulated manner.

### Growth Phenotypes of vps45 Mutant Cells Correlate with Reduced Cellular Levels of Snc v-SNAREs

*VPS45* is a class D *VPS* gene [15]. Class D *VPS* gene products, including Vps45, and the regulatory and catalytic subunits of PI-3 kinase (Vps15 and Vps34) are required for traffic between the TGN and endosomes [19]. The original *VPS* genes, identified through genetic screens for mutants that missort CPY [11], [12] are not essential due to the existence of multiple pathways for delivery of macromolecules to the vacuole [31], [32]. Consistent with this, most *vps* mutants do not show growth defects under normal laboratory conditions. One exception to this are *vps45* mutants [13], [14]. Figure 3A shows that cells lacking Vps45p have significantly longer doubling time, and slower growth rates, than congenic wild-type cells, whereas other class D *vps* mutants do not. This difference in growth phenotype between *vps45* mutant cells and other class D *vps* mutants is also illustrated by the observation that *vps45* mutants display a marked growth defect at 39°C whereas other class D mutants tested do not (Fig. 3B). These observations are surprising, as removal of different components involved in the same transport step would be expected to result in similar phenotypes.

Although not identified in the original *vps* genetic screens, *tlg2* mutants are *vps* mutants in that they missort the vacuolar hydrolase CPY [33]. Like other *VPS* genes, *TLG2* is not essential. Similarly, Vam3, the syntaxin that regulates traffic at the vacuolar membrane is also not essential [34], [35]. In contrast SNAREs required for membrane traffic to the cell surface are essential [36]. As well as participating in endosomal Tlg2-containing SNARE complexes [33], Snc2 is also required for the fusion of secretory vesicles with the plasma membrane for exocytosis [26]. Exocytosis is required for growth, and as such *snc* mutants are inviable [26]. We hypothesized that the growth phenotype of *vps45*Δ cells might be due to their reduced levels of Snc v-SNAREs and took advantage of our finding that expression of the Vps45_L117R_ mutant in *vps45*Δ cells selectively restores the levels of Snc2 (Fig. 2) to test this. Figures 4A and 4B show that expression of Vps45_L117R_ complements the reduced growth rate, longer doubling time and temperature-sensitive growth phenotypes of *vps45*Δ cells as efficiently as wild-type Vps45.

We have also previously characterised a second mutant of Vps45p, Vps45_W244R_, which gives a dominant negative phenotype for sorting of vacuolar hydrolases [7], [27]. The CPY missorting phenotype of this dominant negative mutant is dose-dependent and can be abrogated by co-expression of either wild-type Vps45 or Snc2, but not Tlg2 [7]. This suggests that Vps45_W244R_ disrupts endosomal trafficking by titrating out the v-SNARE Snc2. Expression of Vps45_W244R_ in *vps45*Δ cells does not complement the CPY missorting phenotype observed in *vps45*Δ cells [7], [27]. Figure 4B shows that expression of the mutant Vps45_W244R_ in *vps45*Δ cells partially complements the temperature-sensitive growth phenotype of *vps45*Δ cells, restoring growth at 39°C. Moreover, Figure 4C demonstrates that co-expression of HA-Snc2 from a multicopy plasmid with Vps45_W244R_ fully restores growth to levels seen with Vps45p and Vps45p_L117R_ mutants. These data indicate that the growth defects of *vps45*Δ cells are independent of the CPY missorting phenotype as they can be overcome by increasing Snc2 levels in cells containing a non-functional version of Vps45p.

It is important to remember that SM protein function in SNARE-mediated membrane traffic extends beyond a role in maintaining cellular levels of its binding partners. This is demonstrated by our previous work showing that restoration of Tlg2 levels in *vps45*Δ cells is not sufficient to complement the vacuolar trafficking defects of these cells, and has also been reported for other SM/Syntaxin pairs including syntaxin-1/Munc18-1 [5]. The multiple functions of SM proteins complicates the challenge of understanding the role(s) of this protein family *in vivo* [2]–[4]. The observation that increasing levels of Snc2 complements growth phenotypes of *vps45*Δ cells likely reflects the role of the exocytic SM protein, Sec1p, in regulating Snc-containing SNARE complexes at the plasma membrane, as oppose to Vps45 in the endosomal system v-SNAREs [4]. Further studies will be required to ascertain whether this is the case as alternative/compensatory mechanisms or pathways may complicate genetic analyses *in vivo.* The data presented here indicate that the growth phenotypes of cells lacking Vps45 are not related to the SM proteins function in SNARE complex assembly, but are a secondary consequence of the reduced levels of Snc2 in these cells. Work is currently underway to identify mutant versions of Vps45 that stabilise Tlg2, but not Snc2, and also versions of Snc2 that are not subject to downregulation in the absence of Vps45. Such tools will allow us to address many important questions that arise from the present study, such as what factors determine whether a particular Snc participates in an exocytic or an endosomal SNARE complex.

Here, we have demonstrated that Vps45 plays a role in regulating the cellular levels of, not only its syntaxin (Tlg2) but also its v-SNARE (Snc) binding partners. Furthermore, we have shown that this reduction in cellular Snc levels correlates with the impaired growth phenotypes seen for *vps45* mutant cells. Prior to this study, this had been a puzzling observation since these growth phenotypes are not displayed by cells lacking Tlg2. Growth phenotypes are associated with loss of Vps45 orthologues in other organisms: in addition to endocytic defects, vps-45 mutation in *Caenorhabditis elegans* results in temperature sensitive lethality [37], and RNAi knockdown of AtVPS45 in *Arabidopsis thaliana* results in stunted growth [23]. Further work is required to investigate whether these are due to reduced levels of exocytic v-SNAREs. In addition, this study describes how cells may regulate levels of SNARE proteins as a mechanism to control membrane trafficking pathways.

## Materials and Methods

### Yeast Strains and Plasmids

Yeast strains and plasmids used in this study are listed in Tables 1 and 2. The Class D yeast mutants used, in addition to NOzY1 (Table 1; *vps45*Δ), in Figure 3 are all congenic to SF838-9D (Table 1) and are as follows: *pep12*, SGY39 [38]; *vps15,* mut.2066 [15]; *vps21*, SGY79 [39]; *vps34,* mut.223 [15]. Yeast were grown either in rich media [1% (w/v) yeast extract, 1% (w/v) peptone, 2% (w/v) glucose: YEPD] or standard minimal medium with appropriate supplements [40]. RMY8 was constructed from SEY6210 [11] using pLO2010 as previously described [41]. MSY002 was then constructed from RMY8 using pNOzY13 to disrupt *VPS45* as described [5]. pCOG054 encoding an HA-tagged version of Sncp was constructed using PCR to amplify genomic sequence of *SNC2* with ∼500 bp up stream and ∼300 bp down stream with the following oligonucleotides (156; 5′-TTAATACGAACAAAATAAAAATATG-3′ AND 157; 5′-AAGACGGCCACTAAAACTGATG-3′). The resultant product was cloned into pCR2.1-TOPO (Invitrogen), sequenced and subsequently subcloned (using the *Kpn*I and *Xho*I sites contained within the oligonucleotides) into pRS426 [42]. Site directed mutagenesis was then used to introduced sequence encoding an HA-epitope tag immediately after the start codon of *SNC2*.

### Antibodies

Polyclonal antibodies that specifically recognise the Snc v-SNAREs, Tlg2, Vps45 and Pgk1 and have been described previously [5], [14], [28]. Antibodies against the HA (3F10) epitope tags were from Sigma.

### Immunoblot Analysis of Yeast Cell Lysates

Yeast cells were grown in standard minimal media (SD, lacking amino acids where appropriate for plasmid selection) [40]. 10 OD_600_ equivalents were harvested from cultures in early log phase and vortexed in 200 µl of Laemmli sample buffer in the presence of glass beads (425–600 µm, acid washed). Glass beads were allowed to settle during a 10 min incubation at 65°C and 10 µl of the lysate was loaded per lane of a 10% SDS-polyacrylamide gel. Proteins were next transferred to nitrocellulose for immunoblot analysis with antibodies as indicated. Immunoreactive proteins were visualized by detection with an anti-rabbit/mouse IgG horse radish peroxidase linked secondary antibody as appropriate, and enhanced chemiluminescence (both from Amersham). Protein levels were quantified by densitometry using ImageJ software (NIH) in relation to Pgk1 levels (which was used as a loading control).

### Growth Rate Determination

Yeast were grown for 16 h at 30°C in a rotary shaker (250 rpm), then diluted into fresh medium at OD_600_ = 0.2. OD_600_ was measured every hour until the stationary phase of the growth curve was reached. The doubling time (DT) during logarithmic growth was determined as described by Willett [43]: DT = ln 2×*t*/(ln *b* - ln *a*) (*t* is the time period in hours; *a* is the optical density at the beginning of time period; *b* is the optical density at the end of time period). The specific growth rate (μ) was calculated as described by Harvey [44]: μ = (ln *b* – ln *a*)×2.303/*t*.
